# Sex Differences in the Behavioural Aspects of the Cuprizone-Induced Demyelination Model in Mice

**DOI:** 10.3390/brainsci12121687

**Published:** 2022-12-08

**Authors:** Kelly F. Paton, Sheein Hong, Andrew Biggerstaff, Bronwyn M. Kivell

**Affiliations:** Centre for Biodiscovery, School of Biological Sciences, Victoria University of Wellington, Wellington 6012, New Zealand

**Keywords:** cuprizone, demyelination, motor coordination, anxiety, sex differences, multiple sclerosis

## Abstract

Multiple sclerosis is an autoimmune disease characterised by demyelination in the central nervous system. The cuprizone-induced demyelination model is often used in mice to test novel treatments for multiple sclerosis. However, despite significant demyelination, behavioural deficits may be subtle or have mixed results depending on the paradigm used. Furthermore, the sex differences within the model are not well understood. In the current study, we have sought to understand the behavioural deficits associated with the cuprizone-induced demyelination model in both male and female C57BL/6J mice. Using Black gold II stain, we found that cuprizone administration over 6 weeks caused significant demyelination in the corpus callosum that was consistent across both sexes. Cuprizone administration caused increased mechanical sensitivity when measured using an electronic von Frey aesthesiometer, with no sex differences observed. However, cuprizone administration decreased motor coordination, with more severe deficits seen in males in the horizontal bar and passive wire hang tests. In contrast, female mice showed more severe deficits in the motor skill sequence test. Cuprizone administration caused more anxiety-like behaviours in males compared to females in the elevated zero maze. Therefore, this study provides a better understanding of the sex differences involved in the behavioural aspects of cuprizone-induced demyelination, which could allow for a better translation of results from the laboratory to the clinic.

## 1. Introduction

Multiple sclerosis (MS) is an autoimmune disease of the central nervous system (CNS) involving the inappropriate activation of the immune system, leading to demyelination and neurodegeneration. MS affects approximately 2.8 million people worldwide [1]. The most common form of MS, relapsing and remitting MS, is 2–3 times more common in females than males [2,3,4]. MS can present with a variety of different symptoms depending on the site of the lesion and commonly causes motor deficits, cognitive impairment, bladder and bowel issues, pain, visual disturbances, depression and anxiety [5]. There are current therapies for MS that modulate the immune system which are more successful at treating the relapsing–remitting forms of the disease. However, none restore lost function or repair damaged myelin, and current therapies are ineffective at treating progressive forms of the disease [6].

There is significant interest and demand for developing remyelination therapies that aim to repair damaged myelin and restore function. A commonly used preclinical model to evaluate remyelination is the cuprizone-induced demyelination model [7,8]. Cuprizone is a copper chelator with region-dependent neurotoxicity, that selectively causes oligodendrocyte cell death, resulting in demyelination [9,10]. Traditional methods for observing remyelination following cuprizone-intoxication have been carried out using electron microscopy, histology and immunohistochemistry. However, this does not give quantitative insight into the functional effects of demyelination and remyelination in real-time throughout this model. 

Severe demyelination within the corpus callosum can be exploited in behavioural assays, due to the essential role that the corpus callosum plays in the transfer and communication of information between the hemispheres of the brain, which is especially significant for motor coordination and cognition. Many studies have investigated the motor effects during cuprizone-induced demyelination and have predominantly used the rotarod performance test, with mixed results [11]. Therefore, there is no reliable standard test to evaluate motor deficits in this model. Another common symptom of MS is pain, which is reported in up to 86% of patients; however, despite its frequent occurrence, pain has only been evaluated in approximately 4% of publications involving the cuprizone model [11]. Furthermore, it is important to understand the behavioural deficits of the cuprizone-induced demyelination model in both sexes. Previous studies have shown male and female C57BL/6J mice have similar demyelination patterns and have the same number of oligodendrocytes and glial cells following cuprizone administration [12]. However, 84% of publications using C57BL/6 mice in the cuprizone model used only males and behavioural sex differences have not been systematically compared [11]. Therefore, in the present study, we investigated the differences in motor coordination, mechanical sensitivity, and anxiety-like behaviours in both male and female mice throughout cuprizone-induced demyelination.

## 2. Materials and Methods

### 2.1. Animals

Male (n = 105) and female (n = 77) C57BL/6J mice (8–12 weeks old, 20–30 g) were used for all experimental procedures. Mice were originally sourced from the Jackson Laboratory (Bar Harbor, ME, USA) and were then bred and housed at the Victoria University of Wellington Animal Facility, New Zealand. The mice were group-housed with a maximum of 5 per cage. Male and female mice were housed in separate cages. The facility was set to a 12 h light-dark cycle (lights on from 07:00), with controlled temperature (19–21 °C) and humidity (55%). All mice were habituated to the experimental room for at least 30 min prior to behavioural testing. All experimental procedures were carried out with approval from the Victoria University of Wellington Animal Ethics Committee (approval numbers 28154 and 24383) and in agreement with the New Zealand Animal Welfare Act, 1999.

### 2.2. Cuprizone-Induced Demyelination Model

Mice were given 0.2% or 0.3% (*w*/*w*) cuprizone (bis(cyclohexylidenehydrazide); Santa Cruz Biotechnology, Dallas, TX, USA) in powdered mouse chow (irradiated standard rat and mouse diet, Specialty Feeds, Glen Forrest, WA, Australia) and was administered as 5 g per mouse in a dish placed at the bottom of the cage and replaced each day for 42 days [13,14]. Healthy control mice were given 5 g per mouse of normal powdered food without cuprizone. After day 42 of cuprizone intoxication, mice were given normal pellet food. Water was provided ad libitum.

### 2.3. Black Gold II Staining

On day 42 post-initiation of cuprizone administration, the mice were deeply anesthetised with pentobarbital and transcardially, perfused with 5% heparinised phosphate-buffered saline (PBS; 140 mM NaCl, 2.68 mM KCl, 8.1 mM Na2HPO4, 1.47 mM KH2PO4, pH 7.4) followed by 4% paraformaldehyde (PFA). The brain tissue was dissected and fixed in 4% PFA overnight, and subsequently cryoprotected overnight with 30% sucrose in PBS. Using a brain matrix, the area between bregma +2 to −2 mm was isolated, embedded in cryo-mountant and snap-frozen in isopentane on a bed of dry ice. Coronal 20 µm sections were cut using a Leica CM3050S cryostat microtome and mounted on Superfrost plus slides (Thermo Fisher, Waltham, MA, USA). Black gold II stain (#AG105, Merck Millipore, Burlington, MA, USA; and Histo-Chem Inc., Jefferson, AR, USA) was used according to the manufacturer’s instructions. Each section was imaged at the midline of the corpus callosum using an Olympus BX63 microscope equipped with a 10× objective (Olympus, New Zealand). Using ImageJ software (version 1.52a, National Institutes of Health, Bethesda, MD, USA), the area of staining was measured using thresholding in a region of interest (area of 34,132 µm^2^) and 3–4 sections were analysed per animal.

### 2.4. Mechanical Sensitivity

Mechanical sensitivity was measured using an electronic von Frey aesthesiometer (2390 series, IITC Life Science, Los Angeles, CA, USA) with #7 Supertip filament (up to 7.5 g force). Mice were held in a transparent plastic chamber on a mesh stand for 20 min before beginning the procedure. The filament was applied at a 90° angle to the plantar surface of the hind paw and the pressure increased to elicit a withdrawal response. Each paw was measured in triplicate and averaged per mouse. The mechanical sensitivity measurements were repeatedly tested in the same mice on days 0, 14, 28 and 35 of cuprizone administration.

### 2.5. Horizontal Bars

The triple horizontal bar procedure was based on methods previously described [15,16]. Mice were tested on three horizontal bars of increasing thickness: 2 mm, 4 mm and 6 mm. The bars were 38 cm long and suspended 50 cm above the bench. The mice were placed on the centre of the bar, only grasping with the forepaws. The mouse was timed for 30 s, with the time stopped if the mouse either fell from the apparatus or reached the end of the horizontal bar. If the mouse fell off the bar, it was repeated two more times and the time averaged. A score was given based on the time the mouse remained on the apparatus, as follows: 1 = 0–5 s; 2 = 6–10 s; 3 = 11–20 s; 4 = 21–29 s; 5 = 30 s, or touched the end of the bar. Testing began on the 2 mm bar; if the mouse scored 5, it would progress to the next size bar; if the mouse scored below 5, it would not progress to the next bar. If the mouse progressed to subsequent bars, the scores across all bars were added to give a maximum possible score of 15. The horizontal bars were repeatedly tested on the same mice on days 0, 14, 28 and 35 of cuprizone administration.

### 2.6. Passive Wire Hang

The passive wire hang was based on methods as previously described [17]. Mice were placed with all four paws on a circular metal grid which was inverted and suspended on top of a 50 cm tall plastic chamber with soft bedding to cushion the fall. The latency to fall was measured with a maximum of 75 s. Three trials were carried out for each mouse, with at least 30 s rest between each trial, and the average latency recorded. Passive wire hang was repeatedly tested in the same mice on days 0, 14, 28 and 35 of cuprizone administration.

### 2.7. Motor Skill Sequence Testing

Mice were moved into individually housed motor skill sequence (MOSS) cages (Lafayette Instrument, IN, USA) on day 28 of cuprizone intoxication. The MOSS cages were equipped with a 38-rung training wheel to allow spontaneous running behaviour for 14 days [18], followed by 7 days with a 16-rung complex wheel [19]. Starting on day 35, the mice were given daily intraperitoneal injections of a vehicle solution with a 1:1:8 ratio of dimethyl sulfoxide, Tween-80 and 0.9% saline, respectively. Animal wheel-running activity was continuously recorded from the beginning of the dark cycle (19:00) on day 28. Scurry Activity Monitoring System software (Lafayette Instrument, Lafayette, IN, USA) collected activity data. The cumulative distances travelled on the wheel were analysed in 12 h intervals during the dark cycle on days 28–49. For the maximum velocity values, data were exported in 1 min intervals and the maximum value per dark cycle was identified.

### 2.8. Elevated Zero Maze

The elevated zero maze test was carried out as previously described [20]. The elevated zero maze has an elevated 5 cm wide and 63 cm high circular track divided into four quadrants. Two of these have 16 cm tall dark walls that enclose the quadrants, the other two quadrants are open with a 2 mm clear plexiglass border on each side. Mice naïve to the maze were placed into a tall dark-walled quadrant and allowed to explore the maze freely for 5 min while the activity of the mice was recorded with an overhead camera. The number of times the mouse exited the dark quadrant (defined by all four paws in the open area) and the number of head dips (defined by the head of the mouse passing over the edge of the apparatus, resulting in a postural change) were counted, and the amount of time spent in the open area was determined from the video footage by an observer blinded to the treatment group. The elevated zero maze behaviours were tested between days 33–35 of cuprizone administration.

### 2.9. Marble Burying

The marble burying test was carried out as previously described [20]. Briefly, mice were placed individually in a cage containing 20 marbles on top of 5 cm of bedding. The mice were left for 30 min and a photo was taken of the cage after the mouse was removed. Two blinded observers assessed the images, with marbles counted as buried if less than a third was visible. Marble burying behaviours were tested on day 33 of cuprizone administration.

### 2.10. Z-Score Analysis

Z-scores were calculated as previously described [21]. The test value from an individual mouse (*χ*) was compared to the mean of the control group (*µ*) and the standard deviation of the control group (*σ*), using the formula as follows:

(1) $$Z=\frac{\chi -\mu }{\sigma }$$

For results in which the test value was measured over time, the area under the curve (AUC) was calculated for each mouse to give an individual value to calculate z-score. The directionality of the scores were adjusted to reflect the behavioural effect measured (increase in anxiety, decrease in motor coordination).

### 2.11. Statistical Analysis

GraphPad Prism (version 7.05, GraphPad Software, La Jolla, CA, USA) was used to determine statistical significance. Values are presented as the mean ± standard error of the mean (SEM; for numerical values see descriptive statistics tables Supplementary Tables S1 and S2). The data sets were tested for normality using the D’Agostino and Pearson omnibus normality test. Unpaired t-tests were used to compare between two groups. Comparisons of a single variable between three groups were analysed with one-way ANOVA followed by Bonferroni post-tests. Comparisons of a variable over time were analysed with two-way repeated-measures ANOVA, followed by Bonferroni post-tests. Comparisons were considered significant when *p* < 0.05, and were adjusted for multiple families of comparisons.

## 3. Results

### 3.1. Cuprizone Administration Led to Increased Weight Loss in Male Mice Compared to Females

**Figure 1:**
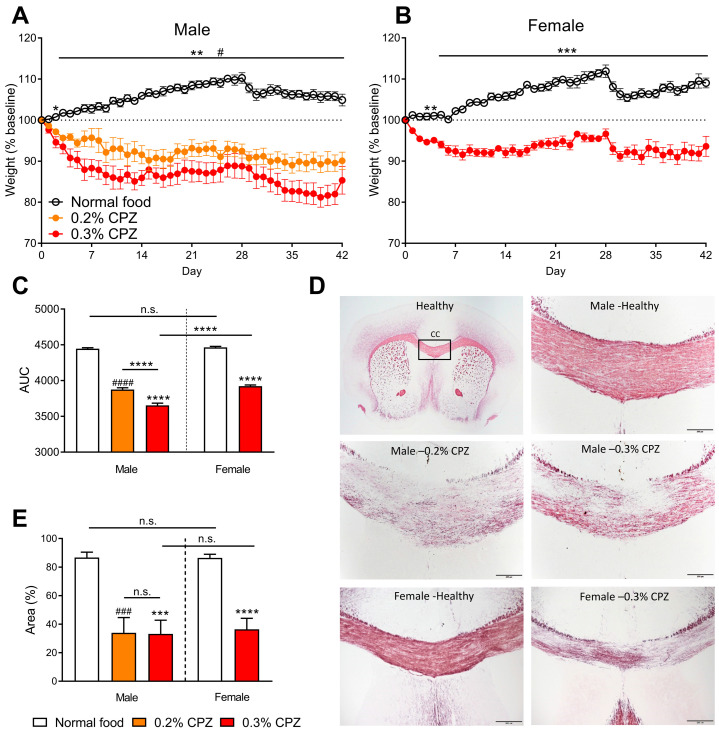
Cuprizone administration led to weight loss and demyelination in male and female C57BL/6J mice. The body weight of the mice was measured daily over the 42 days of cuprizone (CPZ) administration. (**A**) Male and (**B**) female mice administered cuprizone lost weight compared to the normal food diet, n = 8–10. (**C**) Area under the curve (AUC) analysis showed that all groups administered cuprizone lost weight, with the 0.3% cuprizone treatment in males showing more severe weight loss than the 0.2% dose in males and the 0.3% dose in females. (**D**) Representative images showing Black gold II myelin stain taken from day 42 of cuprizone administration. (**E**) Threshold analysis gave the percentage of area stained within a region of interest positioned at the midline of the corpus callosum (CC). In the male and female animals treated with cuprizone, there was a reduction in the area of myelin stained compared to mice given normal food. Analysed in 3–4 sections per animal (averaged), n = 5–10. The scale bar is 200 µm. # *p* < 0.05, ### *p* < 0.001, #### *p* < 0.0001 for 0.2% cuprizone vs. normal food. * *p* < 0.05, ** *p* < 0.01, *** *p* < 0.001, **** *p* < 0.0001 for 0.3% cuprizone vs. normal food, or as indicated. n.s. = not significant. Data presented as mean ± SEM.

Mice were weighed daily to assess the health of the animals (Figure 1A,B). Due to the expected severe disease in the males administered 0.3% cuprizone, we chose to also include a lower 0.2% cuprizone dose in the males only. AUC analysis found that 0.2–0.3% cuprizone administration reduced the weight of both the male and female animals compared to the normal food diet (*p* < 0.0001; Figure 1C). Within the male groups, there was a dose-dependent effect with larger weight loss in the 0.3% cuprizone group compared to the 0.2% cuprizone group (*p* < 0.0001; Figure 1C). There was also a sex difference in the weight loss, with males showing a larger weight loss than females treated with 0.3% cuprizone (*p* < 0.0001; Figure 1C).

### 3.2. Cuprizone Administration Led to Demyelination in the Corpus Callosum

Black gold II stain was used to measure the area of myelin in the corpus callosum from mice on day 42 post-initiation of cuprizone administration (Figure 1D). We found that in both male (*p* = 0.0002 for 0.2% cuprizone and *p* = 0.0001 for 0.3% cuprizone) and female (*p* < 0.0001) mice, there was a significant reduction in the area of staining (Figure 1E). Within the male groups, there was no difference between the 0.2% and 0.3% cuprizone treatments (*p* > 0.9999; Figure 1E). There was no sex difference between the normal food groups (*p* > 0.9999) or in the 0.3% cuprizone treatments (*p* > 0.9999; Figure 1E).

### 3.3. Cuprizone Administration Produced Mechanical Sensitivity in Both Male and Female Mice

**Figure 2:**
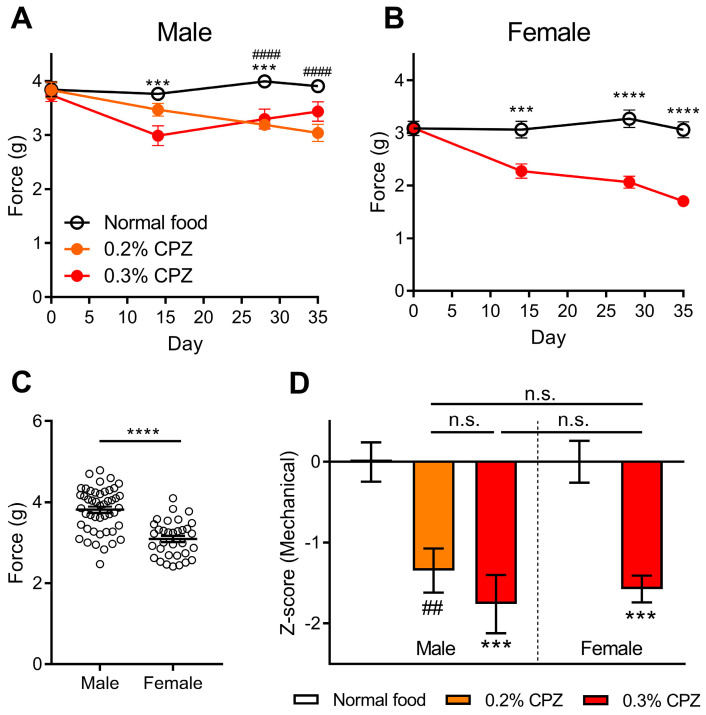
Cuprizone administration decreased mechanical sensitivity threshold in male and female C57BL/6J mice. Mechanical sensitivity was measured using an electronic von Frey aesthesiometer. In (**A**) male and (**B**) female mice, cuprizone (CPZ) administration led to a decrease in the mechanical threshold. Two-way ANOVA with Bonferroni post-tests. (**C**) In a comparison between all mice on day 0, it was found that female mice have a lower threshold than male mice. (**D**) The z-score analysis normalised the results to the corresponding normal food control. All doses of cuprizone led to a decrease in mechanical threshold with no sex or dose-dependent effects. (**A**,**B**) Two-way repeated-measures ANOVA with Bonferroni post-tests. (**C**) Unpaired *t*-test. (**D**) One-way ANOVA with Bonferroni post-tests. ## *p* < 0.01, #### *p* < 0.0001 for 0.2% cuprizone vs. normal food. *** *p* < 0.001, **** *p* < 0.0001 for 0.3% cuprizone vs. normal food, or as indicated. n.s. = not significant. Data presented as mean ± SEM. n = 13–18.

We assessed whether the administration of cuprizone would affect the mechanical sensitivity of the hind paws over the 35 day cuprizone administration period. In both male and female mice, there was a significant decrease in the withdrawal threshold upon the administration of cuprizone, compared to the mice given normal food (Figure 2). The 0.3% cuprizone administration produced mechanical sensitivity from days 14–28 (*p* < 0.001) in males and days 14–35 (*p* < 0.001) in females (Figure 2A,B). The 0.2% cuprizone dose in males increased mechanical sensitivity between days 28–35 (*p* < 0.0001; Figure 2A). We further investigated whether there was an innate sex difference between all mice tested on day 0 (prior to cuprizone administration), and found that females had a lower mechanical threshold compared to males (*p* < 0.0001; Figure 2C). For this reason, we used z-score analysis to normalise the results to the respective normal food controls. The results showed that all mice administered cuprizone had a decrease in the mechanical threshold (*p* < 0.01), with no sex or dose-dependent differences (*p* > 0.9999: Figure 2D).

### 3.4. Effects of Cuprizone Administration on Motor Coordination Were Significantly Greater in Male Mice Compare to Females

**Figure 3:**
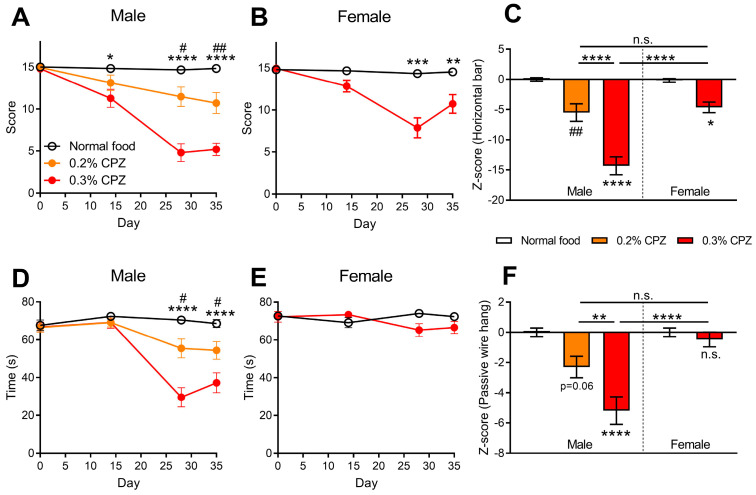
Cuprizone administration decreased motor coordination in C57BL/6J mice. Motor coordination was measured using the horizontal bar test in (**A**) male and (**B**) female mice. Cuprizone (CPZ) administration led to a decrease in the score in both sexes. (**C**) Z-score analysis of the area under the curve data showed a sex and dose-dependent effect, with the 0.3% cuprizone treatment in males decreasing the horizontal bar score more than the other cuprizone treatment groups. The passive wire hang test in (**D**) male and (**E**) female mice showed a deficit in cuprizone-treated male mice but not female mice. (**F**) Z-score analysis showed the 0.3% dose of cuprizone affected males greater than females. (**A**,**B**,**D**,**E**) Two-way repeated-measures ANOVA with Bonferroni post-tests. (**C**,**F**) One-way ANOVA with Bonferroni post-tests. # *p* < 0.05, ## *p* < 0.01, for 0.2% cuprizone vs. normal food. * *p* < 0.05, ** *p* < 0.01, *** *p* < 0.001, **** *p* < 0.0001 for 0.3% cuprizone vs. normal food, or as indicated. Data presented as mean ± SEM. n = 12–18.

Motor coordination was assessed using both the horizontal bar and the passive wire hang tests (Figure 3). For the horizontal bar test, in males, the mice that received cuprizone administration had a decreased motor coordination score between days 14–35 for the 0.3% dose (*p* < 0.05) and days 28–35 for the 0.2% dose (*p* < 0.05; Figure 3A). In the female mice, 0.3% cuprizone only had a significant effect between days 28–35 (*p* < 0.01; Figure 3B). The z-score analysis showed that the most severe deficit was in the males that received 0.3% cuprizone, which was significantly greater than the 0.2% cuprizone males (*p* < 0.0001) and the 0.3% cuprizone females (*p* < 0.0001; Figure 3C).

From the horizontal bar test, the males showed deficits for both concentrations of cuprizone at days 28–35, compared to the mice administered normal food (0.2% cuprizone *p* < 0.05, 0.3% cuprizone *p* < 0.0001; Figure 3D); conversely, the females did not show any changes in the motor coordination score over the 35 days in this model (*p* > 0.05; Figure 3E). The z-score analysis showed that only the 0.3% cuprizone dose in males was significantly different from the normal food control (*p* < 0.0001) and was more severe than the corresponding dose in females (*p* < 0.0001) and the 0.2% cuprizone treatment in males (*p* = 0.0057; Figure 3F).

### 3.5. Cuprizone Administration Decreased Spontaneous Running Wheel Activity

**Figure 4:**
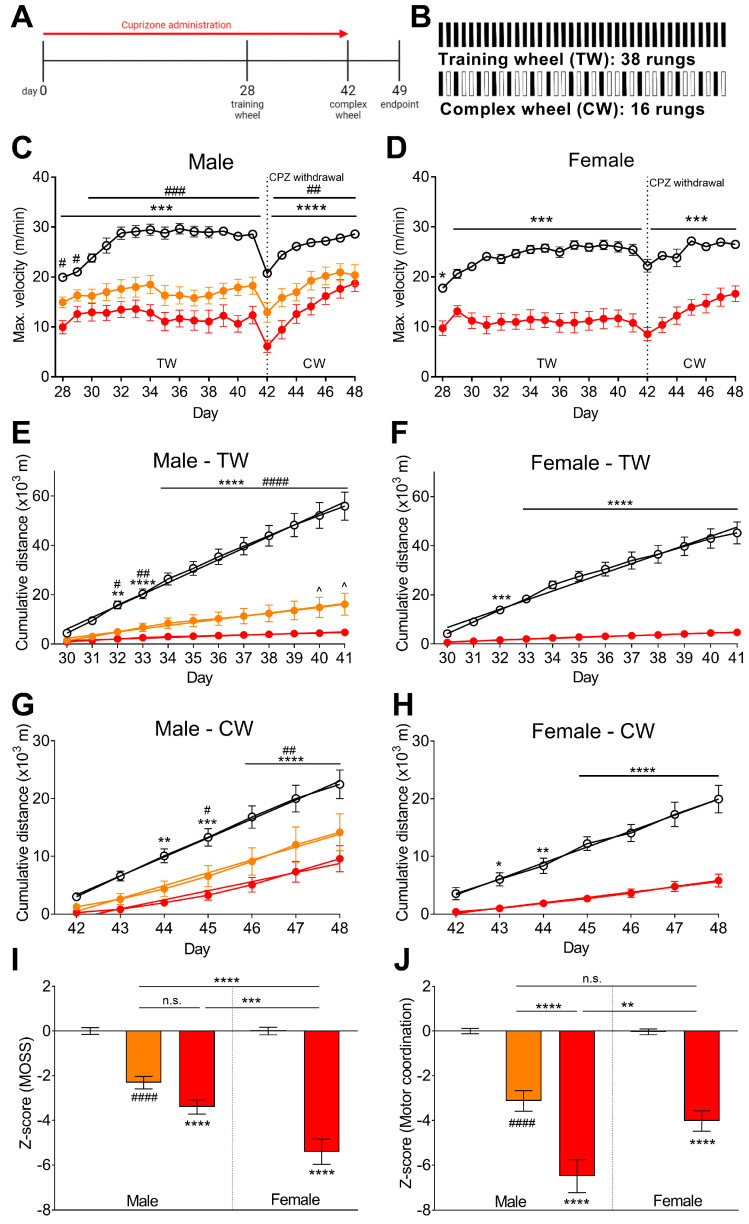
Cuprizone administration decreased complex motor coordination in C57BL/6J mice. (**A**) Timeline of motor skill sequence (MOSS) testing in the cuprizone-induced demyelination model. The training wheel (TW) is used on days 28–42 and the complex wheel (CW) on days 42–49. (**B**) Schematic diagram of MOSS wheel rung pattern, 38 rungs for the training wheel and 16 rungs for the complex wheel. The maximum velocity per day for the length of the experiment in (**C**) males and (**D**) females showed a deficit with cuprizone administration. Cumulative distance travelled was recorded on the (**E**,**F**) training wheel on days 28–41 and the (**G**,**H**) complex wheel on days 42–49. Mice on normal food exercised significantly more than cuprizone-intoxicated mice on the training and complex wheel. (**I**) Z-score analysis for the MOSS testing showed the 0.3% cuprizone affected females more severely than males. However, (**J**) z-score analysis of all measures from MOSS testing, horizontal bar and passive wire hang, overall showed that the 0.3% dose in males had the greatest deficit in motor coordination. (**C**–**H**) Two-way repeated-measures ANOVA with Bonferroni post-tests. (**I**,**J**) One-way ANOVA with Bonferroni post-tests. # *p* < 0.05, ## *p* < 0.01, ### *p* < 0.001, #### *p* < 0.0001 for 0.2% cuprizone vs. normal food. ** *p* < 0.01, *** *p* < 0.001, **** *p* < 0.0001 for 0.3% cuprizone vs. normal food, or as indicated. ^ *p* < 0.05 for 0.2% cuprizone vs. 0.3% cuprizone. Data presented as mean ± SEM. n = 8–10.

The MOSS activity wheel is reported to be a more sensitive method of detecting latent deficits in motor coordination, as it measures spontaneous home-cage activity [19]. We used a standard protocol with two weeks on the training wheel (38 rungs), starting on day 28, and one week on the complex wheel (16 rungs), starting on day 42 when the cuprizone administration was also withdrawn (Figure 4A,B). Cuprizone administration reduced the maximum velocity during the dark cycle on every day measured in males (Figure 4C) and females (Figure 4D).

The cumulative distance was measured for the training wheel and the complex wheel. On the training wheel, all doses of cuprizone in males (Figure 4E) and females (Figure 4F) reduced the running distance from day 32–41. There was a significant difference between the male doses of cuprizone on days 40 and 41 (*p* < 0.05; Figure 4E). For the complex wheel, in males the 0.3% cuprizone dose reduced running distance on days 44–48 (*p* < 0.01) and the 0.2% cuprizone dose on days 45–48 (*p* < 0.05; Figure 4G). For the females, the 0.3% cuprizone dose reduced running distance from days 43–48 (*p* < 0.05; Figure 4H). Similarly, the daily distance run showed significant decreases in the mice administered cuprizone (Supplementary Figure S1).

The calculated z-score incorporated all MOSS behaviours and showed that all doses of cuprizone reduced MOSS behaviours, compared to normal food controls (*p* < 0.0001; Figure 4I). There was no difference between the doses in males (*p* = 0.2038), however, there was a sex difference, with the females administered 0.3% cuprizone showing a greater reduction in behaviour compared to males (*p* = 0.0003; Figure 4I). Finally, we calculated the z-score using the standard models of motor coordination, horizontal bar and passive wire hang, as well as the spontaneous complex motor coordination results from the MOSS activity. The results showed that all doses of cuprizone reduced the z-score (*p* < 0.0001), and there was a sex (*p* = 0.0012) and dose (*p* < 0.0001) dependent effect, with the 0.3% cuprizone male group showing the most severe deficit (Figure 4J).

### 3.6. Cuprizone Administration Increases Anxiety-Like Behaviours in Male Mice but Not in Female Mice

**Figure 5:**
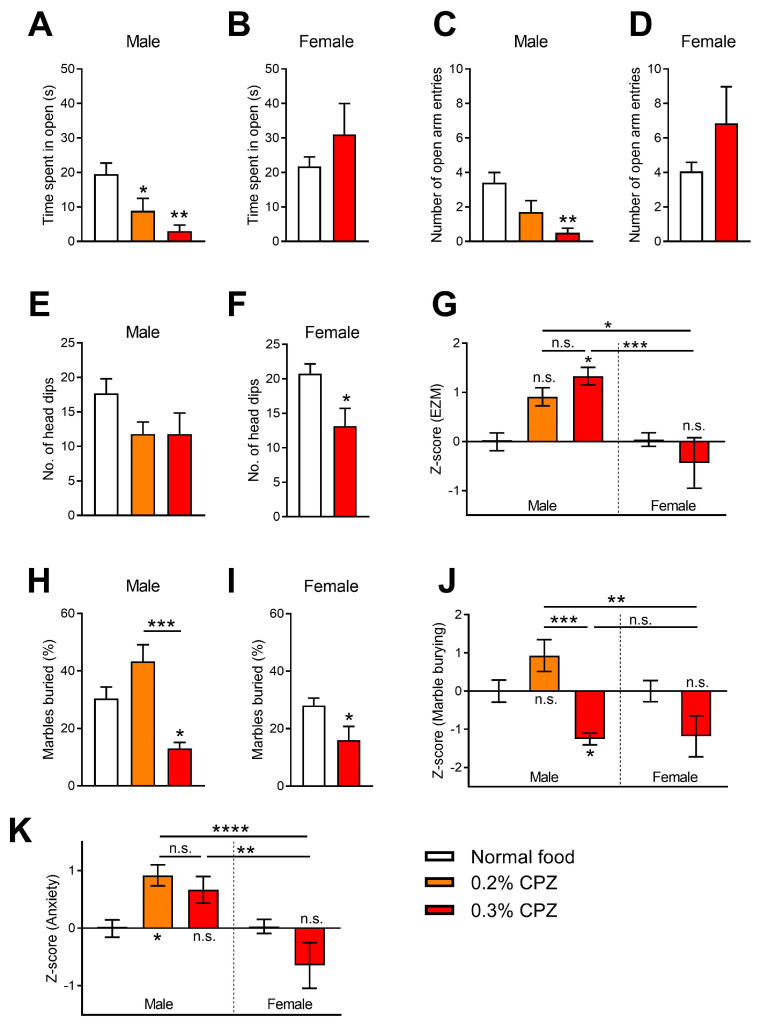
Administration of cuprizone has anxiogenic effects in male mice but not female mice. The elevated zero maze tested mice for 5 min with (**A**,**B**) time spent in the open arm, (**C**,**D**) the number of open arm entries and (**E**,**F**) the number of head dips measured for both sexes. Cuprizone in male mice exhibited a dose-dependent reduction in the number of open arm entries and a reduction in the time spent in the open arms. (**G**) Z-score analysis of all behaviours on the elevated zero maze (EZM) showed that only the male 0.3% cuprizone treatment group had an anxiogenic effect. (**H**,**I**) The marble burying test was used to measure anxiogenic and repetitive–compulsive behaviour in the mice. The 0.3% cuprizone dose reduced the number of marbles buried in both sexes. (**J**) Z-score analysis of the marble burying behaviour confirmed there was no sex difference between the 0.3% cuprizone dose. (**K**) Z-score analysis of the elevated zero maze and marble burying data combined showed an increase in anxiety-like behaviours in male mice treated with cuprizone compared to female mice. Unpaired t-tests for female comparisons and one-way ANOVA with Bonferroni post-tests for the male and z-score comparisons. * *p* < 0.05, ** *p* < 0.01, *** *p* < 0.001, **** *p* < 0.0001. n.s. = not significant. Data presented as mean ± SEM. n = 10–16.

We assessed anxiety-like behaviours in the elevated zero maze, using the time spent in the open arm, the number of open arm entries and the number of head dips as measures. The male mice treated with 0.2–0.3% cuprizone spent significantly less time in the open area (0.2% cuprizone *p* = 0.0353, 0.3% cuprizone *p* = 0.0011; Figure 5A) and had fewer open arm entries (0.3% cuprizone *p* = 0.0015; Figure 5C) compared to healthy mice. In comparison, the female mice did not have altered behaviour in this test compared to the mice that received normal food (open arm time *p* = 0.2940, open arm entries *p* = 0.1743; Figure 5B,D). For the head dips measurements, the male cuprizone groups showed no difference compared to the control (*p* = 0.2657; Figure 5E), whereas the females fed 0.3% cuprizone had a reduction in the number of head dips compared to normal food controls (*p* = 0.0111: Figure 5F). The z-score was calculated to combine all measures for the elevated zero maze that showed males treated with 0.3% cuprizone had increased anxiety-like behaviours compared to normal food controls (*p* = 0.0212), and there was a significant sex difference (*p* = 0.0003; Figure 5G). 

We further investigated the effect of cuprizone administration on marble burying, an indicator of anxiety and repetitive–compulsive behaviours [22,23]. We found that the 0.3% cuprizone dose in both males (*p* = 0.0415; Figure 5H) and females (*p* = 0.0422; Figure 5I) reduced the number of marbles buried, indicating an anxiolytic response. The z-score analysis showed a difference between the 0.2% and 0.3% cuprizone dose in males (*p* = 0.0003) and no difference between the sexes at the 0.3% cuprizone dose (*p* > 0.9999; Figure 5K). Lastly, we calculated a z-score based on the results from the elevated zero maze and the marble burying test (Figure 5K). Only the 0.2% cuprizone male group showed a difference compared to the normal food control (*p* = 0.0473), and the female 0.3% cuprizone group had significantly less anxiety-like behaviours than both the male cuprizone groups (*p* = 0.0013 vs. 0.3% and *p* < 0.0001 vs. 0.2%; Figure 5K).

### 3.7. Cuprizone Administration Had the Most Severe Deficit in Males Administered the 0.3% Dose

**Figure 6:**
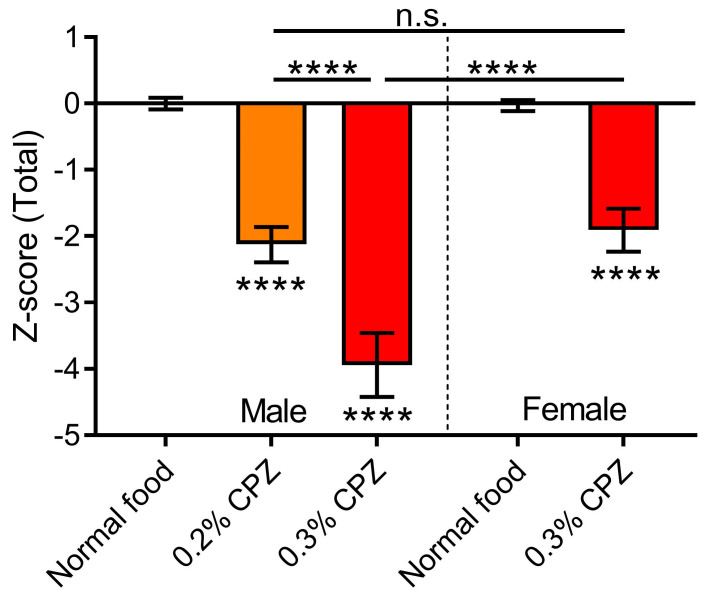
Cuprizone has more severe behavioural deficits in males compared to females. Z-score analysis was used to compare the treatment groups normalised to the healthy control for each sex. The total score was calculated from mechanical sensitivity, horizontal bar, passive wire hang, motor skill sequence, elevated zero maze and marble burying behavioural tests. In males, there was a dose-dependent effect with a more severe deficit in the 0.3% cuprizone group. When compared between sexes, the 0.3% cuprizone dose had more severe behavioural deficits in the males rather than females. One-way ANOVA with Bonferroni post-tests. **** *p* < 0.0001, n.s. = not significant. Stars below the bars indicate comparisons to the corresponding healthy control group. Data presented as mean ± SEM.

Finally, the total z-score was calculated based on all the results from mechanical sensitivity, horizontal bar, passive wire hang, motor skill sequence, elevated zero maze and marble burying behavioural tests (anxiety z-score directionality altered). The results showed that all cuprizone treatment groups were different compared to healthy control mice (*p* < 0.0001; Figure 6). The male 0.3% cuprizone group had the biggest reduction in z-score (*p* > 0.9999 vs. 0.2% cuprizone males; *p* < 0.0001 vs. 0.3% cuprizone females), indicating the greatest change in behavioural functions, whilst the male 0.2% and female 0.3% cuprizone groups were not significantly different from one another (*p* > 0.9999; Figure 6).

## 4. Discussion

In the current study, we have sought to understand the behavioural deficits associated with the cuprizone-induced demyelination model in both male and female mice. A better understanding of behavioural models will allow for a better translation of results from the laboratory to the clinic. Furthermore, understanding the effect of sex on behavioural outcomes will enable the evaluation of novel remyelination drugs that aim to repair damage and improve function. To further examine the data, we combined similar behaviours and used z-score analysis to normalise the results for each sex to the corresponding healthy response, allowing for a direct comparison between the sexes.

We initially measured the body weight of the mice each day to give an overall indication of the health of the animals following cuprizone administration in the food source. All doses of cuprizone in both sexes showed a decrease in body weight over 42 days of cuprizone administration (Figure 1A,B). These findings are consistent with previous studies that have also shown weight loss in mice administered cuprizone [14,24,25,26,27,28,29,30]. The z-score analysis revealed a sex and dose-dependent effect, with the 0.3% cuprizone dose in males showing the greatest weight loss (Figure 1C). The majority of papers with published weight loss data only assessed male mice, however, Stidworthy et al. [29] assessed both sexes with no apparent sex differences. Another paper reported that female mice fed 0.3% cuprizone for 2 weeks had significant weight loss on days 4–14, whereas the male mice only had weight loss on days 8–14 [31], suggesting there may be sex differences in sensitivity to cuprizone. However, this study only assessed 2 weeks of cuprizone administration, whereas the standard acute demyelination model requires 5 to 6 weeks of cuprizone administration to achieve robust demyelination [8].

We then assessed the myelin levels using Black gold II stain to ascertain whether there were sex or dose differences following cuprizone administration (Figure 1D). We found there was significant demyelination in the corpus callosum of both male and female mice on a cuprizone diet (Figure 1E). These results are consistent with previous Black gold II staining in male mice fed 0.2% cuprizone diet for 5 weeks [32] and demyelination in the corpus callosum as measured, using luxol fast blue stain and myelin basic protein (MBP), proteolipid protein (PLP) and myelin oligodendrocyte glycoprotein (MOG) immunolabelling in male mice [25,26,33,34,35] and female mice [36]. We further found that the Black gold II stain did not detect any sex differences in levels of myelin (Figure 1E). The previous literature shows the strain of the mice may be an important factor when studying sex differences, with more severe cuprizone-induced demyelination in males in Swiss [37] and SJL mice [38]; however, in C57BL/6 mice, there was no difference in demyelination or oligodendrocyte numbers between the sexes [12]. Therefore, with no difference in the level of demyelination between the sexes, we further sought to understand the effect of sex on behavioural aspects of demyelination.

A recent analysis found that pain-like behaviours were evaluated in only 4% of publications involving the cuprizone-induced demyelination model [11]. Up to 86% of patients report pain as a symptom of MS [11], with approximately 24–26% characterised as neuropathic pain [39,40]. Therefore, we evaluated changes in mechanical sensitivity, a common characteristic of neuropathic pain, using von Frey filaments. In the experimental autoimmune encephalomyelitis (EAE) model of MS, von Frey filaments have been used to show mechanical allodynia in the early stages of disease progression, before the occurrence of hind limb paralysis [41,42]. We found that both male and female mice showed a decrease in the force needed to elicit a withdrawal response in cuprizone-treated mice (Figure 2A,B). In an electrical stimulation model of neuropathic pain, there was a significant decrease in the neuropathic pain threshold in C57BL/6J mice fed a 0.2% cuprizone diet [43], and female C57BL/6J mice administered cuprizone via oral gavage (400 mg/kg daily) showed a 65% reduction in the cold sensitivity reaction score measured using the acetone test [44]. In contrast, two previous studies used both mechanical and thermal stimuli in male mice administered 0.2% cuprizone, and found no differences in the withdrawal thresholds over 5 weeks [28,45]. Both of these studies used mice of C57BL/6 background and used a dynamic plantar aesthesiometer with a ramp speed of 2 g/s, which produced larger baseline withdrawal responses than the current study (Figure 2C); the study used an electronic von Frey aesthesiometer that is manually applied to the footpad, with the user determining the application speed. We also know that the sex of the experimenter can affect the outcome of pain models [46]. Overall, very few studies have investigated neuropathic pain in the cuprizone-induced demyelination, with a couple showing no difference [28,45], and our study adding to previous work showing that neuropathic pain is associated with cuprizone administration [43,44].

Motor behaviours have frequently been assessed in the cuprizone-induced demyelination model [11]. Evoked locomotor behavioural tests, such as the rotarod performance task, have been used in the cuprizone model with mixed results, either showing deficit (increased frequency of falls or decreased latency to the first fall) [14,25,32,47,48,49,50,51,52,53,54,55,56,57,58,59,60,61] or no difference between cuprizone-fed mice and controls [24,45,62]. We initially used the horizontal bar and the four limbs passive wire hang tests to measure motor coordination and grip strength [15,63]. We found that in male mice there were deficits in both models, whereas female mice only showed a deficit in the horizontal bar test, suggesting sex differences in sensitivity within these tests (Figure 3). This could be due to the larger coordination required for the horizontal bar task, whereas the passive wire hang is a more accurate assessment of strength and fatigue-like behaviours [15,63]. Previous studies in male mice administered cuprizone have found a deficit in a two-limb wire hang test [47], the beam balance performance test [62] and in the walking ladder task [28], but no deficit in the four-limbs passive wire hang test [45]. In female mice administered cuprizone, there was a deficit in the four limbs passive wire hang [51] and grip strength [64]. Overall, it appears that there is a deficit caused by cuprizone in these motor coordination models.

Evoked tests of motor coordination may not sensitively detect latent motor deficits, whereas more complex tasks, such as the MOSS activity wheel, may provide a more accurate assessment by measuring spontaneous home-cage activity [19]. Furthermore, the use of the complex wheel, with rungs irregularly spaced, allows for a more sensitive assessment of the bimanual motor coordination that is dependent on an intact corpus callosum [65]. Using the MOSS apparatus, we found robust deficits in the training and complex wheel running distance in both sexes (Figure 4). This is consistent with previous studies that found decreased maximum running velocity, running distance and run duration during 0.2% cuprizone administration [18,19,66,67,68]. Interestingly, when we used the z-scores to compare behaviours, the MOSS data was the only comparison that showed females to be more severely affected than males (Figure 4I). Of the previous studies using MOSS in the cuprizone model, both male [19,68] and female C57BL/6 mice [18] had been used in separate publications, however, the sexes had not been directly compared.

Another common symptom of MS is anxiety, which has been assessed in the cuprizone-induced demyelination model with mixed results. The elevated plus maze has been used frequently, with cuprizone-fed animals showing either higher levels of anxiety with less time in the open area [69,70], no difference [71,72,73], or an anxiolytic effect with more time in the open arm [74,75,76,77]. Instead of the elevated plus maze, we used the elevated zero maze to assess anxiety-like behaviours. The elevated zero maze removes the ambiguous centre of the plus maze, which accounts for 13–30% of the test time [78], therefore, allowing for more robust comparisons. We found the cuprizone-fed male mice showed anxiety-like behaviours, however, the female mice only showed a reduction in the number of head dips, with no change in the time spent in the open arm (Figure 5A–G). One disadvantage to the use of the elevated zero maze is that it relies on the mice to be able to explore the maze, and as we have shown, the mice administered cuprizone have reduced motor coordination. However, this does not account for the female mice as they do not have reduced time in the open arm but show the most severe deficit in the MOSS test.

The effect of cuprizone has been tested in other preclinical models of anxiety, including open field and light-dark tests. The majority of papers using the open field test showed that a cuprizone diet caused increased anxiety with decreased time or distance in the centre zone [55,70,72,75,76,79,80,81,82]; conversely, whereas, others found increased time in the centre [25,83] or no difference [35,84]. Using the light-dark test, mice fed a cuprizone diet spent less time in the light box, which is correlated to increased anxiety [70,85]. We used the marble burying test to further add to the depth of the literature in this area, as an indicator of anxiety and repetitive–compulsive behaviours [22,23]. We found that mice administered 0.3% cuprizone did not show anxiety and buried fewer marbles; however, there was a difference between the two doses in males, with the males administered 0.3% cuprizone burying fewer marbles (Figure 5H,I). We have not found any other literature assessing marble burying behaviour in mice on a cuprizone diet. However, one paper used mice without a major structural component of myelin, PLP1, showing that the PLP1-null mice buried fewer marbles than the wildtype controls [86]. Overall, the literature appears to be mixed on the occurrence of cuprizone-induced anxiety, which was also reflected in our results, with 0.3% cuprizone in males producing anxiety in the elevated zero maze but not the marble burying test. In the z-score analysis for all anxiety-like behaviours, we did find a sex difference with the females producing fewer anxiety-like behaviours (Figure 5K).

One of the limitations of the cuprizone-induced demyelination model is ensuring the mice are delivered a consistent dose of cuprizone. By mixing the cuprizone with the powdered food source, the dose is dependent on the amount consumed by each mouse and reliant on consistent mixing of the formulation. There are now pellets containing cuprizone available, however, reports that the pellets produce both less consistent [62,87] and more consistent [88] demyelination than the powdered chow formulation vary study to study. Furthermore, there have been studies to administer the cuprizone via oral gavage to ensure a consistent dose was delivered [89,90], however, this requires more handling and stress on the animals, and may lead to oesophageal perforation with daily administration for up to 6 weeks.

In the current study, we have assessed neuropathic pain behaviours by using mechanical sensitivity thresholds (Figure 2); however, further aspects of neuropathic pain, such as sensitivity to cold, and other forms of pain require further study within the cuprizone-induced demyelination model. The analysis from Sen et al. [11] found that 29–86% of MS patients had pain symptoms, however, only 4% of papers using the cuprizone-induced demyelination model assessed pain-like behaviours. Similarly, both cognitive impairments and depressive symptoms are common amongst MS patients [11], and further investigation is required to understand whether these deficits are present in the cuprizone-induced demyelination model.

## 5. Conclusions

We set out to understand the sex differences within the behavioural deficits associated with cuprizone-induced demyelination in mice. Cuprizone administration caused significant demyelination in the corpus callosum, however, there were no sex differences in the levels of demyelination. We found that the deficits were more severe in males for weight loss, horizontal bar, passive wire hang and elevated zero maze tests. The mechanical sensitivity measurements showed no sex differences, whereas the MOSS combined behaviours showed that females had a more severe deficit in motor coordination. When behavioural data were combined to give an overall z-score, the males fed a 0.3% cuprizone diet had the most severe deficit (Figure 6). Therefore, this study provides a better understanding of the sex differences involved in the behavioural aspects of cuprizone-induced demyelination. Previously, the majority of studies have been carried out in male mice, so an understanding of the comparison to females would allow for a better translation of results from the laboratory to the clinic.

## Data Availability

The data presented in this study are available on request from the corresponding author.

## Supplementary Materials

The following supporting information can be downloaded at: https://www.mdpi.com/article/10.3390/brainsci12121687/s1, Figure S1: Daily distance travelled during motor skill sequence testing in mice administered cuprizone. Table S1: Descriptive statistics for weight change, demyelination, motor skill sequence, elevated zero maze and marble burying tests; Table S2: Descriptive statistics for mechanical sensitivity, horizontal bars and passive wire hang tests.

## Abbreviations

AUC, area under the curve; EAE, experimental autoimmune encephalomyelitis; MOSS, motor skill sequence; MBP, myelin basic protein; MOG, myelin oligodendrocyte glycoprotein; MS, multiple sclerosis; PLP, proteolipid protein.
