# Supplementary material for: Sex Differences in the Behavioural Aspects of the Cuprizone-Induced Demyelination Model in Mice
Source: Brain Sci. 2022 Dec 8;12(12):1687. doi: 10.3390/brainsci12121687 (PMC9775311; doi:10.3390/brainsci12121687)
Supplement: Supplementary file 1 [file brainsci-12-01687-s001.zip › brainsci-2033319-supplementary.pdf]

		n	mean	SEM
**Weight change – Area under the curve**				
**Male**	Normal food	10	4446	14.28
	0.2% CPZ	8	3875	25.47
	0.3% CPZ	8	3653	31.71
**Female**	Normal food	8	4465	12.72
	0.3% CPZ	8	3922	17.3
**Demyelination in the corpus callosum – Area (%)**				
**Male**	Normal food	5	86.73	3.739
	0.2% CPZ	5	33.95	10.70
	0.3% CPZ	5	33.20	9.588
**Female**	Normal food	10	86.43	2.573
	0.3% CPZ	5	36.40	7.701
**Motor skill sequence (MOSS) – Z-score**				
**Male**	Normal food	10	0.000106	0.159
	0.2% CPZ	8	-2.314	0.282
	0.3% CPZ	8	-3.404	0.322
**Female**	Normal food	8	-0.00115	0.168
	0.3% CPZ	8	-5.409	0.567
**Elevated zero maze – Time spent in open**				
**Male**	Normal food	10	19.50	3.23
	0.2% CPZ	10	8.90	3.58
	0.3% CPZ	10	3.00	1.78
**Female**	Normal food	16	21.75	2.77
	0.3% CPZ	13	31.00	9.00
**Elevated zero maze – Number of open arm entries**				
**Male**	Normal food	10	3.40	0.60
	0.2% CPZ	10	1.70	0.67
	0.3% CPZ	10	0.50	0.27
**Female**	Normal food	16	4.06	0.53
	0.3% CPZ	13	6.85	2.12
**Elevated zero maze – Number of head dips**				
**Male**	Normal food	10	17.70	2.11
	0.2% CPZ	10	11.80	1.74
	0.3% CPZ	10	11.80	3.04
**Female**	Normal food	16	20.75	1.41
	0.3% CPZ	13	13.15	2.56
**Marble burying – Marbles buried (%)**				
**Male**	Normal food	12	6.08	0.80
	0.2% CPZ	15	8.67	1.16
	0.3% CPZ	10	2.60	0.43
**Female**	Normal food	13	5.62	0.51
	0.3% CPZ	15	3.20	0.95

Supplementary Table S1. Descriptive statistics for weight change, demyelination, motor skill sequence, elevated zero maze and marble burying tests. CPZ = cuprizone; SEM = standard error of the mean.

			Day 0		Day 14		Day 28		Day 35	
		n	mean	SEM	mean	SEM	mean	SEM	mean	SEM
**Mechanical sensitivity – Force (g)**										
**Male**	Normal food	17	3.84	0.14	3.76	0.10	3.99	0.11	3.91	0.08
	0.2% CPZ	17	3.83	0.15	3.47	0.12	3.19	0.10	3.04	0.16
	0.3% CPZ	13	3.74	0.12	2.99	0.18	3.30	0.18	3.44	0.18
**Female**	Normal food	15	3.09	0.14	3.07	0.16	3.27	0.17	3.06	0.15
	0.3% CPZ	18	3.10	0.09	2.27	0.14	2.07	0.11	1.70	0.09
**Horizontal bars – Score**										
**Male**	Normal food	12	15.0	0.0	14.8	0.2	14.7	0.2	14.8	0.1
	0.2% CPZ	17	14.9	0.1	13.1	0.9	11.5	1.2	10.7	1.3
	0.3% CPZ	15	14.8	0.2	11.3	1.1	4.80	1.1	5.20	0.8
**Female**	Normal food	15	14.8	0.1	14.7	0.2	14.3	0.4	14.5	0.4
	0.3% CPZ	18	14.9	0.1	12.8	0.7	7.89	1.2	10.7	1.1
**Passive wire hang – Time (s)**										
**Male**	Normal food	12	67.6	2.8	72.3	1.6	70.5	1.9	68.6	2.2
	0.2% CPZ	17	66.5	2.5	69.1	2.3	55.4	5.0	54.4	4.7
	0.3% CPZ	14	66.7	2.6	69.2	3.1	29.6	5.1	37.3	5.3
**Female**	Normal food	12	72.7	1.4	69.2	2.7	74.0	1.0	72.3	1.8
	0.3% CPZ	15	72.2	2.8	73.3	1.0	65.2	3.4	66.5	3.3

Supplementary Table S2. Descriptive statistics for mechanical sensitivity, horizontal bars and passive wire hang tests. CPZ = cuprizone; SEM = standard error of the mean.

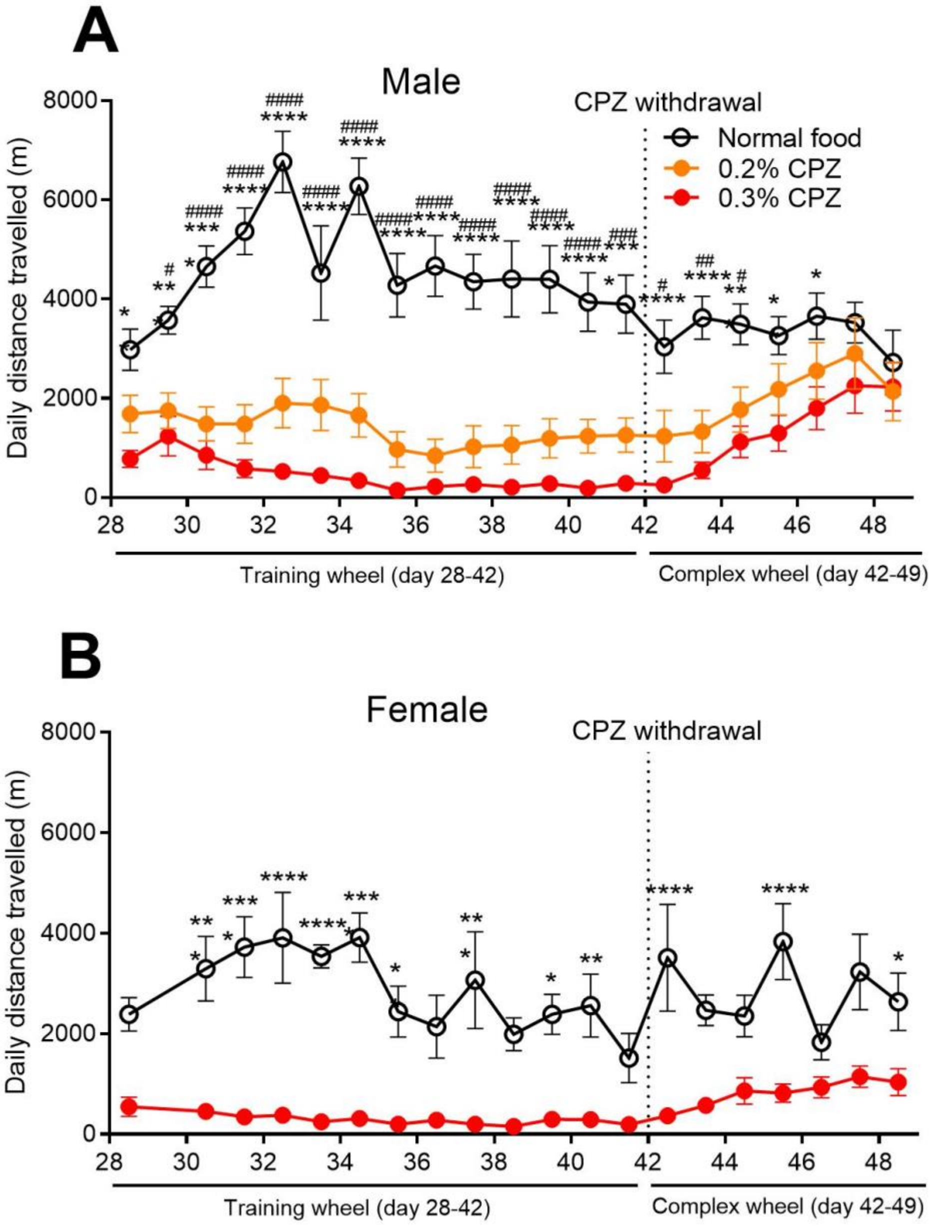

Supplementary Figure S1. Daily distance travelled during motor skill sequence testing in mice administered cuprizone.

Daily distance travelled for (A) male and (B) female mice treated with cuprizone (CPZ) on the training and complex wheels. Administration of cuprizone to both sexes reduced the distance travelled compared to animals given normal food. Two-way repeated measures ANOVA with Bonferroni post-tests. #p<0.05, ##p<0.01, ###p<0.001, ####p<0.0001 for 0.2% cuprizone vs normal food. *p<0.05, **p<0.01, ***p<0.001, ****p<0.0001for 0.3% cuprizone vs normal food. Data presented as mean ±SEM. n=8-10.
